# Individualization of Hematopoietic Stem Cell Transplantation Using Alpha/Beta T-Cell Depletion

**DOI:** 10.3389/fimmu.2019.00189

**Published:** 2019-02-11

**Authors:** Emelie Rådestad, Mikael Sundin, Johan Törlén, Sarah Thunberg, Björn Önfelt, Per Ljungman, Emma Watz, Jonas Mattsson, Michael Uhlin

**Affiliations:** ^1^Division of Transplantation Surgery, Department of Clinical Sciences, Intervention and Technology (CLINTEC), Karolinska Institutet, Stockholm, Sweden; ^2^Division of Pediatrics, Department of Clinical Sciences, Intervention and Technology (CLINTEC), Karolinska Institutet, Stockholm, Sweden; ^3^Hematology/Immunology/HSCT Section, Astrid Lindgren Children's Hospital, Karolinska University Hospital, Stockholm, Sweden; ^4^Cell Therapy and Allogeneic Stem Cell Transplantation, Karolinska University Hospital, Stockholm, Sweden; ^5^Department of Oncology-Pathology, Karolinska Institutet, Stockholm, Sweden; ^6^Science for Life Laboratory, Department of Applied Physics, Royal Institute of Technology, Stockholm, Sweden; ^7^Division of Hematology, Department of Medicine, Karolinska Institutet, Stockholm, Sweden; ^8^Department of Clinical Immunology and Transfusion Medicine, Karolinska University Hospital, Stockholm, Sweden; ^9^Division of Medical Oncology and Hematology, Princess Margaret Cancer Centre and University of Toronto, Toronto, ON, Canada

**Keywords:** αβ T-cell depletion, γδ T-cells, allogeneic hematopoietic stem cell transplantation, stem cell booster, donor lymphocyte infusion, graft manipulation, CliniMACS

## Abstract

Allogeneic hematopoietic stem cell transplantation (HSCT) is associated with several potentially lethal complications. Higher levels of CD3+ T-cells in the graft have been associated with increased risk of graft-versus-host disease (GVHD), but also beneficial graft-versus-leukemia effect and reduced infections. To tackle post-transplant complications, donor lymphocyte infusions have been used but with an increased risk of GVHD. To reduce this risk, we performed depletion of αβ T-cells and treated 12 patients post-HSCT suffering from infections and/or poor immune reconstitution. The αβ T-cell depleted cell products were characterized by flow cytometry. The median log depletion of αβ T-cells was −4.3 and the median yield of γδ T-cells was 73.5%. The median CD34+ cell dose was 4.4 × 10^6^/kg. All 12 patients were alive 3 months after infusion and after 1 year, two patients had died. No infusion-related side effects were reported and no severe acute GVHD (grade III-IV) developed in any patient post-infusion. Overall, 3 months after infusion 11 out of 12 patients had increased levels of platelets and/or granulocytes. In conclusion, we describe the use of αβ T-cell depleted products as stem cell boosters with encouraging results.

## Introduction

Allogeneic hematopoietic stem cell transplantation (HSCT) is commonly used today to treat hematological malignancies, inborn errors of metabolism and immunodeficiencies (1). The overall aim is to replace the entire hematological system of the patient with that of a healthy donor. Treatment procedures and conditioning regimens have continuously evolved over the past decades improving the outcome for treated patients worldwide (2). However, there are still serious complications associated with the treatment including graft rejection, poor immune reconstitution and graft-versus-host disease (GVHD). In addition, the patients undergoing HSCT are at the time of transplantation heavily immunocompromised also giving rise to problems associated with opportunistic infections, caused by for example Epstein-Barr virus (EBV) and Cytomegalovirus (CMV), which can become life-threatening for the patient (3). In the case of hematological malignancies, minor histoincompatibilities are desired to achieve the potent graft-versus-leukemia (GVL) effect in order to eliminate residual tumor cells. However, this can result in the donor immune cells also attacking healthy recipient tissues giving rise to GVHD. T-cells are discussed to be the main effectors of GVHD development as complete depletion of T-cells from the graft have been shown to prevent development of GVHD. However, depleting all T-cells greatly increases the risk of relapse and infectious complications and is therefore not an optimal approach (4, 5). The balance of obtaining optimal GVL and minimal GVHD is clearly delicate and complicated.

T-cells can be divided into two major and distinctly different categories based on their T-cell receptor (TCR) expression. Approximately 95% of circulating peripheral blood T-cells express an αβ TCR while around 5% express a γδ TCR. These two categories of T-cells differ in their development, maturation, activation and function (6). γδ T-cells are considered to be on the border between innate and adaptive immunity as they beyond the traditional specific T-cell activation pathway, can become activated in a non-specific MHC/HLA-independent manner by for example toll-like receptors, NK-cell receptors and phosphoantigens. Recent years have highlighted the importance of γδ T-cells in both anti-viral and anti-tumor immunity. In contrast, αβ T-cells are activated only in the highly specific MHC/HLA-dependent manner and give rise to problems with incompatibility in the setting of allogeneic HSCT, causing serious complications such as graft rejection and GVHD. However, they are important in the protection and induction of specific responses against for example virus reactivations commonly observed after allogeneic HSCT (7, 8).

Re-transplantation or infusion of a donor lymphocyte infusion (DLI) or stem cell booster can be options for patients who display poor immune reconstitution or who suffer from infectious complications post-HSCT. However, all approaches also increase the risk of unwanted GVHD. In order to maximize the beneficial effects of infusing more donor cells and minimize the risk of developing GVHD, depletion, or purification of specific cell types in the infusion product have been made with varying results. Purification of CD34+ stem cells (9), depletion of CD3+ T-cells (10), depletion of CD19+ B-cells, depletion of CD45RA+ naïve T-cells (11) and numerous more strategies have been or are currently under trial. We and few others (12–14) have chosen to selectively deplete αβ T-cells, leaving γδ T-cells in the cell product to retain their many beneficial functions. In a pilot trial, we treated five patients with αβ T-cell depleted stem cell boosters to treat problems with poor immune reconstitution and infections with promising results (12). Now, depletion of αβ T-cells in combination with HSCT is becoming more common (15). In this retrospective analysis, we describe 12 infusions of αβ T-cell depleted cell products given as stem cell boosters. The results are encouraging and αβ T-cell depleted cell products seem to be a viable option after HSCT as well as being part of the graft management in patients at risk of increased GVHD and with non-malignant disease.

## Materials and Methods

### Patient Characteristics

This retrospective study was approved by the Regional Ethical Review Board of Stockholm, Sweden (DNR 2017/2421-31/1) and in accordance with the Declaration of Helsinki. A total of 12 patients received αβ T-cell depleted cell products as stem cell boosters (Table 1). Primary indication for receiving a stem cell booster was primary graft failure (GF) or poor graft function (PGF) with subsequent poor immune reconstitution, infectious problems, and/or mixed chimerism (Table 1, see Supplemental Table 1 for definitions). Major infectious problems are provided in Supplemental Table 2. One pediatric patient (patient 5) had received an αβ T-cell depleted graft as re-transplantation and 7.5 months later received an additional αβ T-cell depleted cell product as a booster (described in the current study). Overall, four out of the 12 patients had underlying non-malignant diseases while remaining 8 had been treated with HSCT for malignant disease (Table 1). Two patients (patient 10 and 12) also received a CD34+ positively selected product along with the αβ T-cell depleted cell product to further increase the number of CD34+ cells given to the patient (Table 2). All analysis was performed retrospectively.

**Table 1:** Patient characteristics and outcome parameters post-infusion of αβ T-cell depleted cell products.

**Patient ID**	**1**	**2**	**3**	**4**	**5**	**6**	**7**	**8**	**9**	**10**	**11**	**12**
**ORIGINAL HSCT**												
Gender	F	F	F	M	M	M	M	F	M	F	F	M
Age	20	59	43	53	3	35	33	69	20	43	56	62
HSCT indication	SCA	AML	ALL	FL	OP	ALL	AMN	CMML	AML	AML	MF	CMML
Status R/D CMV	+/+	+/–	–/–	+/+	–/–	+/–	+/–	+/+	+/+	+/+	+/+	+/–
Status R/D Toxo	+/–	+/–	–/–	+/–	–/–	–/–	–/–	–/–	–/–	–/–	–/–	+/+
Status R/D EBV	+/+	+/+	+/+	+/+	+/+	+/+	+/–	+/–	+/+	+/–	+/+	+/+
Status R/D HSV1	–/–	–/+	+/–	–/–	–/+	+/–	+/+	–/+	+/+	+/–	–/–	–/–
Primary graft	BM	BM	BM	PBSC	PBSC	PBSC	BM	PBSC	BM	BM	BM	BM
Conditioning	MAC	RIC	MAC	RIC	RIC	MAC	MAC	RIC	RIC	MAC	MAC	MAC
Donor type	SIB	MUD	MUD	MUD	MUD	MUD	MUD	MUD	Haplo	Haplo	Haplo	Haplo
**αβ** **T-CELL DEPLETED INFUSION PRODUCT**												
Infusion type	Boost	Boost	Boost	Boost	Boost	Boost	Boost	Boost	Boost	Boost	Boost	Boost
Preconditioning	N/A	N/A	N/A	N/A	N/A	N/A	N/A	N/A	N/A	N/A	N/A	N/A
Infusion indication^*^	PGF	PGF+Inf	PGF+Inf	PGF+Inf	PGF	PGF	PGF	PGF	PGF	GF	GF	PGF+Inf
Product source	PBSC	BM	PBSC	PBSC	PBSC	PBSC	PBSC	PBSC	PBSC	PBSC	PBSC	PBSC
Day of infusion	+193	+134	+196	+176	+226	+169	+76	+236	+105	+33	+78	+504
Chimerism status at infusion	DC	DC	DC	DC	MC	DC	DC	DC	DC	MC	DC	DC
ACUTE GVHD STATUS^*^												
Grade at infusion	0	1	0	2	0	0	0	0	0	0	0	0
Grade (max)	0	2	3	3	1	2	3	1	2	2	2	2
Time max grade (pre/post-infusion)	N/A	Pre	Pre	Pre	Post	Pre	Pre	Pre	Pre	Post	Pre	Pre/post
TRANSFUSION DEPENDENCY^*^ **(ERYTHROCYTES/PLATELETS)**												
Pre-infusion	E/P	E/P	E/P	E/P	–/–	E/P	E/P	E/–	E/P	E/P	E/–	E/P
Post-infusion	–/–	–/–	E/P	E/P	–/–	–/–	–/–	–/–	E/–	–/–	E/–	P/–
G-CSF TREATMENT^*^												
Pre-infusion	Yes	Yes	No	No	No	No	No	No	Yes	Yes	Yes	Yes
Post-infusion	No	No	No	No	No	No	No	No	No	No	No	Yes
**SURVIVAL STATUS POST** **αβ** **T-CELL DEPLETED INFUSION**												
+3 months	Alive	Alive	Alive	Alive	Alive	Alive	Alive	Alive	Alive	Alive	Alive	Alive
+6 months	Alive	Alive	†	Alive	Alive	Alive	Alive	Alive	Alive	Alive	Alive	Alive
+1 year	Alive	Alive	†	Alive	Alive	Alive	Alive	Alive	Alive	Alive	Alive	†

* *According to definitions in Supplementary Table 1. HSCT, hematopoietic stem cell transplantation; F, female; M, male; SCA, sickle cell anemia; AML; acute myeloid leukemia; ALL; acute lymphatic leukemia; FL, follicular lymphoma; OP, osteopetrosis; AMN, adrenomyeloneuropathy; CMML, chronic myelomonocytic leukemia; MF, myelofibrosis; BM, bone marrow; PBSC, peripheral blood stem cells; RIC, reduced intense conditioning; MAC, myeloablative conditioning; SIB, sibling; MUD, matched unrelated; Haplo, haplo-identical; DC, donor chimerism; MC mixed chimerism; N/A, not applicable; PGF, poor graft function; Inf: infection(s); GF: primary graft failure; GR, graft rejection; GVHD, graft-vs.-host disease; E, erythrocyte transfusion dependent; P, platelet transfusion dependent; G-CSF, granulocyte-colony stimulating factor; †, deceased*.

**Table 2:** Characteristics of αβ T-cell depleted cell products (target fractions).

**Patient ID**	**1**	**2**	**3**	**4**	**5**	**6**	**7**	**8**	**9**	**10**	**11**	**12**
Patient weight (kg)	73	71	65	70	16	65	67	77	82	58	58	81
**ORIGINAL PRODUCT**												
αβ T-cells (×10^6^/kg)	133.0	5.5	365.0	245.0	1370.6	372.9	227.0	209.7	200.7	272.5	394.7	110.4
γδ T-cells (×10^6^/kg)	9.3	0.2	4.7	11.1	89.1	12.8	9.8	4.8	6.9	38.3	12.9	13.5
**TARGET FRACTION**												
Volume (mL)	427	323	312	355	322	846	660	281	372	360	316	205
TNC dose (x10^8^/kg)	24.3	2.6	31.1	15.1	27.0	7.3	3.9	3.9	4.0	4.4	5.0	3.7
Lymph (%)	30.0	3.1	4.9	28.3	10.3	17.9	11	10.7	25	24.7	33.8	15.0
Lymph (×10^6^/kg)	99.7	1.1	23.5	64.3	273.6	136.9	43.0	41.7	99.4	110.2	169.0	55.6
CD3+ (% of lymph)	20.0	16.8	9.3	15.3	10.5	6.3	8.5	7.5	4.9	32.6	5.9	22.2
CD3+ (×10^6^/kg)	20.0	6.1	2.2	9.8	28.7	7.8	3.7	3.1	4.9	35.9	10.0	12.3
αβ T-cells (% of CD3+)	0.4	2.8	1.4	1.0	0.3	0.4	0.8	0.5	0.1	0.02	0.1	0.5
αβ T-cells (×10^4^/kg)	6.7	0.5	3.0	11.5	5.0	3.1	2.9	3.6	0.3	0.8	0.8	0.09
γδ T-cells (% of CD3+)	98.2	88.1	97.3	92.2	99.1	98.6	98.0	99.1	99.5	99.5	99.5	91.6
γδ T-cells (×10^6^/kg)	19.9	0.2	2.1	11.1	28.5	7.7	3.6	3.1	4.8	35.6	9.9	11.3
CD19+ (% of lymph)	48.4	49.3	24.5	56.3	58.5	62.3	54.2	44.9	58.1	38.7	52.5	73.9
CD19+ (×10^6^/kg)	48.3	0.6	5.8	36.2	160.1	84.6	23.3	18.7	57.7	42.6	88.7	41.1
CD16+/56+ (% of lymph)	29.1	27.6	62.6	27.3	25.6	24.7	28.0	46.4	37.0	31.0	40.0	14.0
CD16+/56+ (×10^6^/kg)	29.0	0.3	14.7	17.6	70.0	33.6	12.0	19.3	36.8	34.1	67.6	7.8
CD34+ (%)	1.8	1.3	1.2	1.7	0.7	0.5	1.7	1.2	0.4	0.5	0.7	0.5
CD34+ (×10^6^/kg)	5.5	0.4	5.2	3.6	18.3	3.6	7.4	4.7	1.7	5.3 ^**X**^	4.0	2.4 ^**X**^
**DEPLETION EFFICIENCY**												
Yield γδ T-cells (%)	97.2	84.4	44.2	101	31.9	60.2	36.4	65.1	70.3	92.9	76.7	83.5
Log depl of αβ T-cells	−3.2	−4.5	−3.7	−3.3	−4.4	−4.1	−3.9	−3.8	−4.8	−4.5	−4.7	−5.1

X *Patient 10 and 12 also received a CD34-selected product, (patient 10: 79 mL product with 86% purity; patient 12: 42 mL product with 97.0% purity), total CD34 dose for both products (αβ T-cell depleted and CD34-selected target fractions) is presented. Abbreviations: lymph, lymphocytes; TNC, total nucleated cells; depl, depletion*.

### CliniMACS αβ T-Cell Depletion

Depletion of αβ T-cells was performed as previously published by our group (12) and according to the original setup by Handgretinger et al. (14, 16). Cells were obtained as peripheral blood stem cells (PBSCs, *n* = 11) or from bone marrow (*n* = 1). The single-day apheresis procedure was performed using the Spectra Optia apheresis system (Terumo BCT, Inc., Lakewood, CO, USA) after 4 days of stem cell mobilization in which the donor received daily administration of recombinant human granulocyte colony-stimulating factor (G-CSF, filgrastim, Amgen, Thousand Oaks, CA, USA) at a dose of 10 μg/kg. More details on the apheresis procedure can be found in an earlier publication from our center by Wang et al. (17). Post-collection processing the following day included negative depletion of αβ T-cells using the CliniMACS system (Miltenyi Biotech, Bergisch Gladbach, Germany) as previously published (14) with the exception that no B-cell depletion was performed in our setup. The depletion procedure was performed at room temperature and all CliniMACS products were purchased from Miltenyi Biotech. Briefly, cells were washed with CliniMACS buffer followed by 5 min incubation with human immunoglobulin (Privigen, CSL Behring GmbH, Marburg, Germany). CliniMACS TCR α/β-Biotin was added with a subsequent incubation for 30 min on a rocker. After one wash, CliniMACS Anti-Biotin Reagent was added and incubated for 30 min. After washing and cell counting, αβ T-cells were depleted from the cell product on a CliniMACS device using the Depletion 3.1 program. Original fraction, target and non-target fraction were analyzed by flow cytometry for quality control. Specifications for the booster target product included <5 × 10^4^ αβ T-cells/kg and >4 × 10^6^ CD34+ cells/kg. Cells from target and non-target fractions were also frozen and stored at −196°C for subsequent analysis.

### Isolation of Peripheral Blood Mononuclear Cells

Blood samples from 9 out of 12 patients were collected in heparinized tubes at median 28 days post-infusion (range 18–34 days). Peripheral blood mononuclear cells (PBMCs) were isolated using density gradient centrifugation with Lymphoprep (1.077 g/cm^2^, Fresenius Kabi, Oslo, Norway) for 20 min at 800 × g followed by two washes with PBS. The cells were frozen in 1640 RPMI medium (Thermo Scientific, Waltham, MA, USA) with 10% human heat-inactivated AB serum (Karolinska University Hospital, Stockholm, Sweden) and 10% CryoSure dimethyl sulfoxide (WAK-Chemie Medical GmbH, Steinbach/Ts, Germany) and were stored at −196°C until analysis.

### Characterization of γδ T-Cell Subsets and Other Immune Cell Types by Flow Cytometry

Target fractions/PBMCs were thawed in 1640 RPMI with 10% AB serum and washed twice with PBS. Cells were stained with titrated antibodies provided below for 20 min at 4°C. Cells were centrifuged at 700 × g for 4 min and washed once with PBS. Viability staining was performed with 7AAD (BD Biosciences, San Jose, CA, USA) according to the manufacturer. Samples were acquired on a BD Canto with BD FACSDiva v.7.0 software (BD Biosciences). Data was analyzed in FlowJo v.10.1 (Becton, Dickinson and Company, Franklin Lakes, NJ, USA). Gating strategy included singlets, live cells, lymphocytes, CD3 and further subpopulations. Proportions of B-cells and NK-cells were analyzed from CD3- cells, based on expression of CD19 and CD56/CD16 respectively. T-cells were defined as CD3+ and subsets of T-cells was based on expression of CD4, CD8, CCR7, CD45RO, αβ, γδ, *etc*. All γδ subsets were gated from total (CD3+) T-cells. Regulatory T-cells (Tregs) were gated on CD4+ T-cells and were defined as CD25^high^CD127^−/low^.

### Antibody Specification Used for Extracellular Staining of Target Fractions and PBMCs

Target cell product composition was analyzed with a panel including: fluorescein isothiocyanate (FITC)-conjugated anti-TCRVδ1 (TS8.2, ThermoFisher Scientific), anti-TCRVδ2 (B6, BioLegend, San Diego, CA, USA), anti-TCRVγ9 (B3, BioLegend), anti-TCRVα24 (6B11, BioLegend), anti-TCRγδ pan (IMMU510, Beckman Coulter, Fullerton, CA, USA), anti-TCRαβ (T10B9.1A-31, BD Biosciences), anti-TCRαβ (REA652, Miltenyi), anti-CD25 (M-A251, BD Biosciences), anti-CD158b (CH-L, BD Biosciences), anti-IgM (JES3-9D7, BD Biosciences), anti-CCR6 (G034E3, Biolegend); phycoerythrin (PE)-conjugated anti-CD127 (HIL-7R-M21, BD Biosciences), anti-CD16 (3G8, BD Biosciences), anti-CD20 (DCN46, BD Biosciences); anti-CD161 (HP-3G10, Biolegend); PE-CF594-labeled anti-CD197/CCR7 (150503, BD Biosciences); PE-Cyanine7 (PE-Cy7)-conjugated anti-CD3 (SK7, BD Biosciences); allophycocyanin (APC)-conjugated anti-CD19 (HIB19, BD Biosciences); anti-CD45RO (UCHL1, BD Biosciences), anti-CD56 (NCAM16.2, BD Biosciences), anti-CCR9 (112509, R&D systems, Minneapolis, MN, USA); APC-Cy7-conjugated anti-CD8 (SK1, BD Biosciences), anti-TCRvα 7.2 (3C10, Biolegend); Brilliant Violet (BV) 421-conjugated anti-CD27 (M-T271, BD Biosciences); and V500-conjugated anti-CD4 (RPA-T4, BD Biosciences).

PBMC composition was analyzed with a more extensive panel that included, beyond the antibodies above, FITC-conjugated anti-CD28 (CD28.1, BD Biosciences), anti-CD69 (FN50, BD Biosciences), anti-CD107a (MEM25, BD Biosciences), anti-CD152/CTLA-4 (A3.4H2.H12, LifeSpan Biosciences, Seattle, WA, USA), anti-CD94 (HP-3D9, BD Biosciences); PE-conjugated anti-TCRγδ (REA591, Miltenyi); PE-Cy7 conjugated anti-CD158b (DX27, BioLegend); APC-conjugated anti-CD159/NKG2A (REA110, Miltenyi); APC-H7-conjugated anti-IgD (IA6-2, BD Biosciences); BV 421-conjugated anti-CD39 (TU66, BD Biosciences) and anti-CD56 (NCAM16.2, BD Biosciences).

### Statistics and Calculations

Log depletion was calculated as: log(# of αβ T-cells in start product/# of αβ T-cells in end product). The yield in percentage was calculated as: (total count of γδ T-cells in target fraction / total count γδ T-cells in original fraction) × 100.

Response in terms of platelet, granulocyte and lymphocyte count was analyzed statistically using the non-parametric Friedman test to identify a difference over time. Results were considered significant if *p* < 0.05. *Post hoc* analysis included paired comparisons between infusion day and counts at 3, 6, 9, and 12 weeks post-infusion, respectively, using Wilcoxon signed-rank test. Significance levels were set to *p* < 0.05 (^*^), *p* < 0.01 (^**^), and *p* < 0.001 (^***^). Data was analyzed and graphed using Prism 7 (Graphpad, San Diego, CA).

## Results

### αβ T-Cell Depletion of Bone Marrow and Peripheral Blood Stem Cells

**Figure 1:**
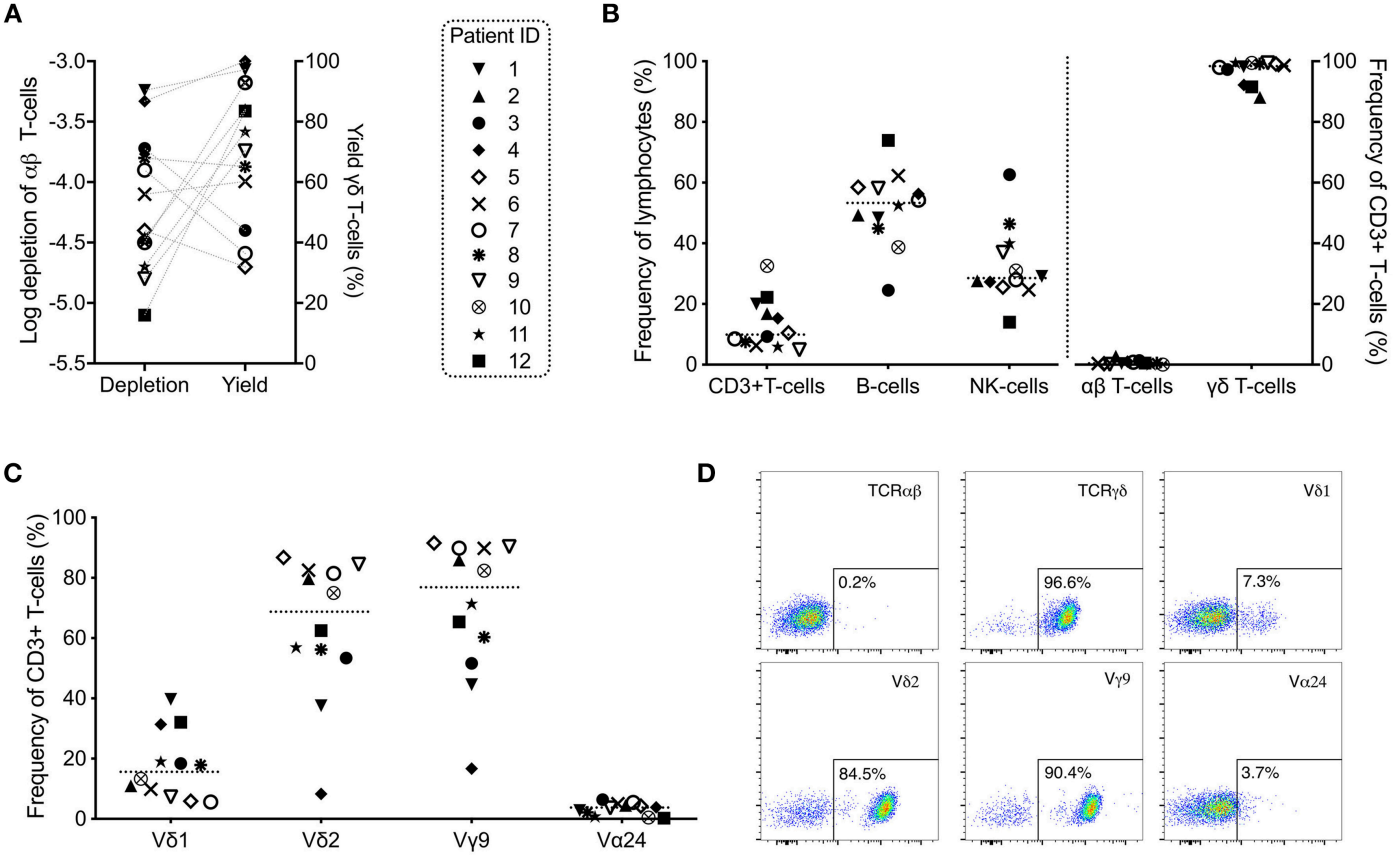
Characteristics of αβ T-cell depleted grafts used as stem cell boosters (*n* = 12). **(A)** Log depletion of αβ T-cells and yield (recovery) of γδ T-cells in target fractions infused into patients (connected with dashed lines). **(B)** Analysis of cell subsets of interest in the αβ T-cell depleted cell products with **(C)** more detailed analysis of γδ subsets and invariant NKT-cells post-depletion gated on total T-cells. **(D)** Representative example plots of T-cells with αβ T-cell receptor (TCR), γδ TCR and γδ subsets are shown from patient 9 (all plots gated on total CD3+ T-cells).

Bone marrow and PBSCs were depleted of αβ T-cells using a CliniMACS device between 3 and 48 h after harvesting for use as stem cell boosters (*n* = 12). The log depletion of αβ T-cells varied between −3.2 and −5.1 (median −4.3) and the yield of γδ T-cells between 31.9 and 101% (median 73.5%) (Figure 1A, Table 2). No significant correlation between high yield and low log depletion could be observed (Figure 1A). When analyzing the αβ T-cell depleted cell products for various subsets of lymphocytes, CD3+ T-cells comprised of 4.9–32.6% (median 9.9%) of all lymphocytes. A clear majority of cells gated on CD3+ T-cells expressed the γδ TCR (88.1–99.5%, median 98.4%). Only 0.02–2.8% (median 0.5%) of the CD3+ T-cells expressed the αβ TCR (Figure 1B, Table 2). CD3- cells consisted mainly of a variation of CD19+ B-cells (24.5–73.9%, median 53.4%) and CD16+/56+ NK-cells (14.0–62.6%, median 28.6%) (Figure 1B, Table 2). The vast majority of γδ T-cells expressed Vδ2 (8.3–86.8%, median 68.8%) and/or Vγ9 (16.7–91.6%, median 76.9%). Vδ1 was expressed by 5.6–39.7%, median 15.6% of total T-cells (Figure 1C) and Vα24 was expressed by a small proportion T-cells (0.2–6.4%, median 3.8%) known as invariant NKT-cells. Representative plots on αβ/γδ T-cells and γδ subsets from the αβ T-cell depleted PBSC booster given to patient 9 are shown in Figure 1D.

### PBMC Characterization 3–5 Weeks Post-infusion

**Figure 2:**
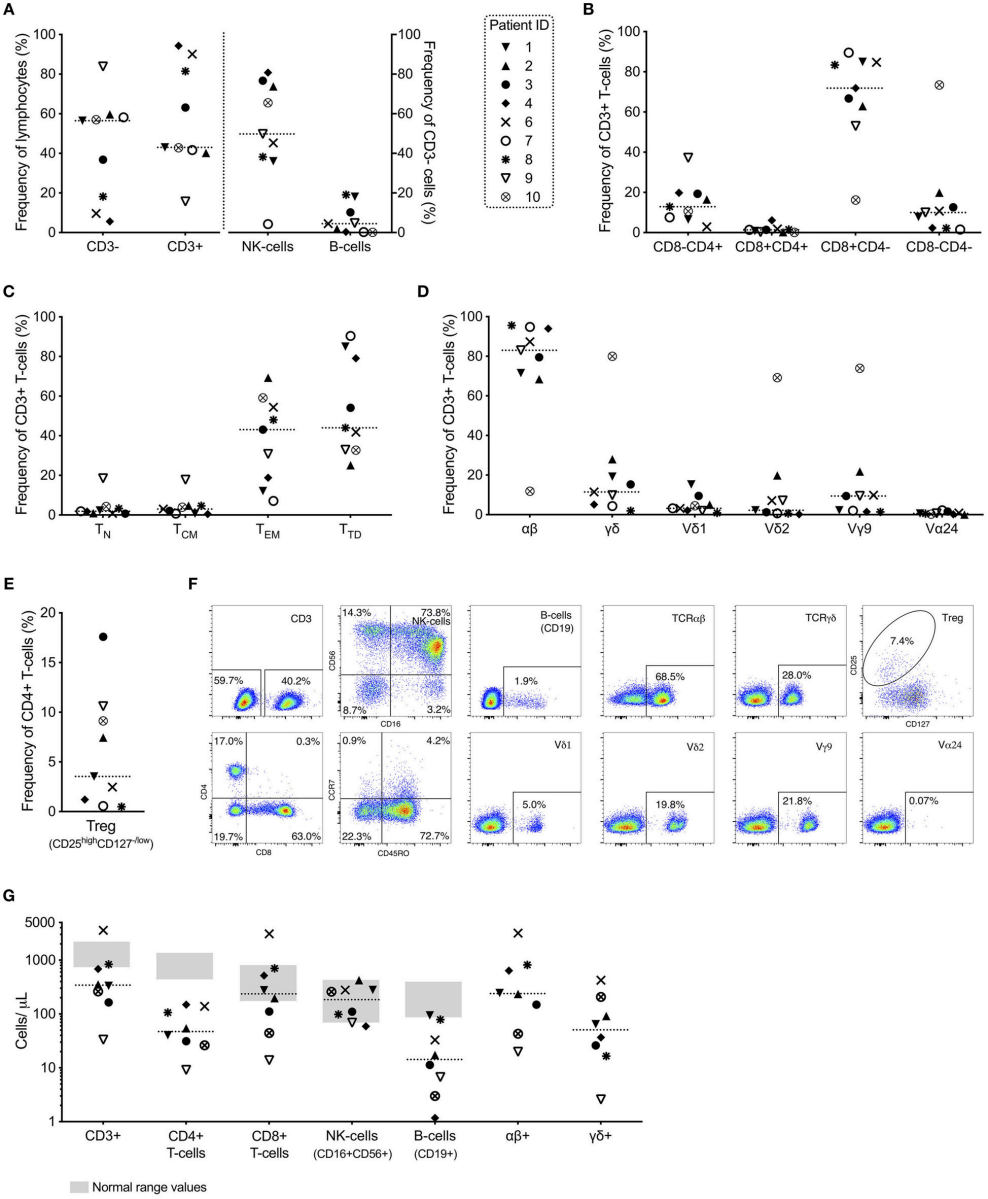
Characterization of peripheral blood mononuclear cells 3–5 weeks (*n* = 9) after infusion of αβ T-cell depleted stem cell booster. **(A)** Major lymphocyte populations: T-cells (CD3+), B-cells (CD19+) and NK-cells (CD56+CD16+); **(B)** CD4 and CD8 T-cell subsets; **(C)** memory/maturation status of total (CD3+) T-cells; naïve (T_N_, CCR7+CD45RO-), central memory (T_CM_, CCR7+CD45RO+), effector memory (T_EM_, CCR7-CD45RO+) and terminally differentiated (T_TD_, CCR7-CD45RO-); **(D)** αβ T-cells, γδ T-cells and subsets of γδ T-cells and invariant NKT-cells (all gated on total T-cells); and **(E)** regulatory T-cells (CD25^high^CD127^−/low^ on CD4+ T-cells) are presented along with **(F)** representative example plots from all indicated populations from patient 2. **(G)** Absolute numbers of T-, B- and NK-cells (with gray areas representing the normal range determined for healthy adult patients) and αβ+ and γδ+ cells, respectively (*n* = 8). Absolute numbers were calculated by multiplying the frequency of the subsets (gated from total alive cells) with the white blood count determined at 4 weeks post-infusion of αβ T-cell depleted cell product.

Retrospective analysis of lymphocytes isolated from peripheral blood from 9 patients 3–5 weeks post-infusion showed a large spread in the proportion and composition of T-cells (Figure 2). Of all lymphocytes, the proportion of CD3+ T-cells varied from 15.7–94.3% (median 43.0%). CD3- cells (median 56.5%) consisted mostly of CD56+CD16+ NK-cells (4.3–80.8%, median 49.8%) and to a less degree of CD19+ B-cells (0.1–19.1%, median 4.5%) (Figure 2A). The majority of T-cells were CD8+ (median 71.9%) and an effector memory (CCR7-CD45RO+) or terminally differentiated memory (CCR7-CD45RO-) phenotype (median 43.1 and 44.0%, respectively) (Figures 2B,C). The majority of T-cells were at this time αβ T-cells (median 83.0%, range 11.8–95.6%) and the proportion of γδ T-cells varied from 1.9–80.0% (median 11.5%) (Figure 2D). The large range was primarily caused by patient 10 who had a high proportion of T-cells expressing the γδ TCR (80.0%) at this time point. Similar to the infusion product, Vγ9 was the most abundant γδ subset among patients at 3–5 weeks post infusion (1.4–73.9%, median 9.4%).

Of all T-cells, median 3.2% comprised of the Vδ1 subset (range 0.9–15.3%), 2.2% of the Vδ2 subset (0.2–69.2%) and limited presence of the Vα24 subset was observed in peripheral blood of these patients (median 0.6%, range 0.1–2.2%) (Figure 2D). Of the CD4+ T-cells, regulatory T-cells (defined as CD25^high^CD127^low/−^) ranged from 0.5–17.6% (median 3.5%) (Figure 2E). Representative plots from PBMCs from patient 2 are shown in Figure 2F.

Absolute numbers of major lymphocyte subsets showed that 8 out of 9 patients 3–5 weeks post-infusion had normal levels of NK-cells (median 185 cells/μL, range 59–425 cells/μL) (Figure 2G). Normal reference values are presented in Supplementary Table 3. Four out of 8 patients also had normal levels of CD8+ T-cells (median 237 cells/μL, range 14–3,080 cells/μL), however all patients had below normal levels of CD4+ T-cells (median 47 cells/μL, range 9–149 cells/μL) resulting in deficient levels of total T-cells for majority of the patients (median 344 cells/μL, range 33–3,590 cells/μL). Also, the levels of B-cells were limited and below normal range for all but one patient (median 14 cells/μL, range 1–95 cells/μL) (Figure 2G).

### Leukocyte and Platelet Recovery After Infusion of αβ T-Cell Depleted Cell Product

Prior to infusion of αβ T-cell depleted products, 11 out of 12 patients were dependent on transfusions of erythrocytes and/or platelets (Table 1). Post-infusion (day +30 until day +180), only two patients were still in need of both erythrocytes and platelets and three patients were dependent on either erythrocytes or platelets. Six out of 12 patients were prior to infusion requiring G-CSF support. Post-infusion, G-CSF support could be discontinued in all patients except one at latest +30 days post-infusion (Table 1).

**Figure 3:**
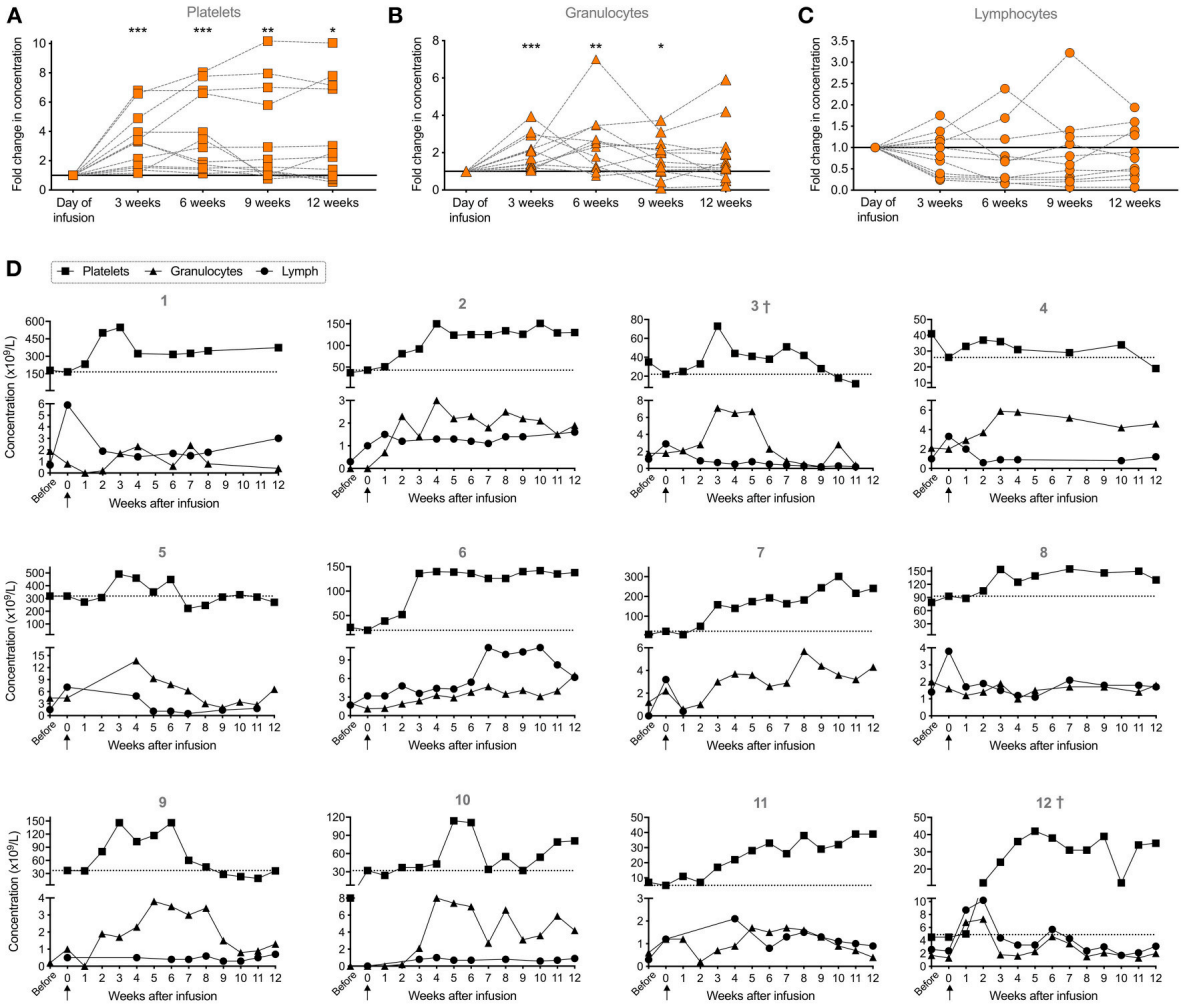
Clinical parameters (platelet, granulocyte and lymphocyte concentrations in peripheral blood) of patients (*n* = 12) before, at day of infusion and 12 weeks post-infusion of αβ T-cell depleted stem cell booster. **(A)** Relative fold change in concentration of platelets, **(B)** granulocytes, and **(C)** lymphocytes at 3, 6, 9, and 12 weeks post-infusion. For patients who did not have a reported count for these specific time points, counts from ±1 week were used. Concentration at the day of infusion was set to 1 (indicated by the line) to follow the relative development. Wilcoxon signed-rank test was used to determine paired statistical differences between each time point and the day of infusion. Significance levels were set to *p* < 0.05 (*), *p* < 0.01 (**), and *p* < 0.001 (***). **(D)** Individual development of the three cellular compartments in each patient (*n* = 12) where week 0 indicates time point of infusion are presented. Dashed line represents platelet concentration at time point of infusion. ^*†*^indicates deceased patients at 12 months post-infusion (*n* = 2, patient 3 died at 4 months and patient 12 died at 8.5 months post-infusion).

To assess the immune reconstitution, platelet, granulocyte and lymphocyte counts were followed until 3 months after infusion (Figure 3). Normal reference values are provided in Supplemental Table 3. To follow the development, the change in concentration was calculated relative to the concentration reported at the day of infusion (Figures 3A–C). The platelet concentration was significantly increased at 3, 6, 9, and 12 weeks post-infusion (*p* < 0.001 for 3 and 6 weeks, *p* = 0.005 for 9 weeks and *p* = 0.027 for 12 weeks, all compared to the day of infusion). After 3 months, the platelet count had increased in 8 out of 12 patients given stem cell boosters. For all patients, median was 106 × 10^9^/L with a range of 12–374 × 10^9^/L at 3 months post-infusion. In two patients no net effect could be seen (patient 5 and 9) and in two patients there was even a decrease (patient 3 and 4) (Figure 3D).

Regarding the development of granulocytes, there was a significant increase in granulocyte count after 3, 6, and 9 weeks post-infusion (*p* < 0.001, *p* = 0.003, and *p* = 0.032, respectively), however at 12 weeks this effect was abolished (Figure 3B). Overall, at 12 weeks post-infusion, granulocyte count varied between patients (range 0.4–6.6 × 10^9^/L) who overall had a median of 2.0 × 10^9^/L. Eight out of 12 patients who received an αβ T-cell-depleted stem cell booster had a beneficial effect on their granulocyte count, even reaching a normal granulocyte count (>1.6 × 10^9^/L), at 12 weeks post-infusion. However, two individuals showed a decrease in granulocyte counts (patient 1 and 3) while two patients (patient 8 and 11) did not change over 3 months (change ≤ 0.2 × 10^9^/L) (Figure 3D).

There was no difference (*p* = ns) regarding changes in lymphocyte counts at 3, 6, 9, or 12 weeks post-infusion (Figure 3C). In 4 out of 11 patients after infusion of the αβ T-cell depleted booster, there was an increase in lymphocyte count (Figure 3D). For one patient (patient 7), no data was available after 1 week. In only 1 out of the 11 evaluated patients, the lymphocyte count was within normal range (>3.5 × 10^9^/L) after 3 months post-infusion (patient 6). For all patients, median lymphocyte count was 1.6 × 10^9^/L, ranging from minimum value of 0.7 to highest 6.2 × 10^9^/L. In two individuals, there was no difference (≤0.3 × 10^9^/L) in lymphocyte count after 3 months (patient 10 and 11) while in five individuals there was a decrease in the lymphocyte count (patient 1, 3, 4, 5, and 8). However, four out of the five patients with a decreased lymphocyte count at 12 weeks (except patient 3), had increased lymphocyte counts when comparing to their count before infusion rather than day of infusion (Figure 3D).

### Infectious Resolution, GVHD, and 1 Year-Status

Ten out of 12 patients had infectious problems at the time point of infusion (Supplemental Table 2). At 3 months post-infusion, 5 out of 10 patients had cleared their major infectious problems completely (including problems with severe mucositis, CMV, *Aspergillus*, adenovirus and local *Staphylococcus aureus*). Three out of 5 patients having CMV-associated problems had cleared the infection at 3 months. Two patients did not have any major infectious complications at the time of infusion of αβ T-cell depleted products and this continued at 3 months post-infusion (Supplemental Table 2).

In total, 11 out of 12 patients developed acute GVHD sometime during their treatment but only three developed their max grade post-infusion of αβ T-cell depleted cell product. Of these three patients, one patient developed grade I and two patients developed grade II (one of which had a re-activation of the acute grade II GVHD he had prior to infusion). Importantly, none of the patients developing acute GVHD grade III during their treatment (*n* = 3) developed the max grade post-infusion. No other infusion-related side effects were noted in any of the patients.

Patient 3 died 4 months after infusion due to a fungal-related cerebral hemorrhage. Patient 12 died at 8.5 months post-infusion due to multiple infections and multi-organ failure. Thus, at 12 months, 10 out of 12 patients were still alive.

## Discussion

In this study, we report the successful use of αβ T-cell depleted cell products as stem cell boosters after HSCT in patients with poor graft function, primary graft failure and/or infectious complications. Overall, the specific role and involvement of the boosters on these parameters cannot be known. Patients in need of stem cell boosters after HSCT generally have a poor prognosis. Studies using non-manipulated grafts as boosters show only an overall survival (OS) of ≤55% (18, 19). Positive selection of CD34+ cells improves the clinical outcomes to some degree (9, 20). In spite of the limited number of patients, our results with 10 out 12 patients (83.3%) surviving more than 1 year are very promising. The majority of patients dependent on transfusion of erythrocytes and/or platelets and G-CSF treatment became post-infusion independent of these supportive measures. Patients with non-malignant disease do not potentially benefit from GVHD through an increase in the GVL effect. This patient group (4 out of 12 patients in our cohort) is therefore especially suitable for receiving αβ T-cell depleted cell products. Depleting αβ T-cells will reduce the risk of GVHD (1), and at the same time increase the number of CD34+ stem cells/kg able to be infused into the patient. This increased CD34+/CD3+ ratio can be achieved with a low dose or absent preconditioning which also will reduce the risk of developing additional complications in these patients.

It remains to be elucidated on the role of the remaining cells in the αβ T-cell depleted cell products. The enrichment of γδ T-cells appears to be important in the management of opportunistic pathogens and offer the desired GVL effect without increasing symptoms for GVHD. Several studies have shown that patients who develop increased numbers of donor-derived γδ T-cells following haploidentical or partially mismatched HSCT have a significant increased leukemia-free survival and OS (8, 21). In addition, γδ T-cells seem to have a positive impact on other factors of HSCT. Ongoing studies have shown their potency in decreasing both engraftment time and incidence of bacterial infections after HSCT (22). After HSCT, increased frequencies and function of γδ T-cells are associated with a protective role against CMV reactivation and disease (7). In accordance with this, several studies have shown increased expansion and cytotoxic function of CMV-reactive γδ T-cells in the peripheral blood of patients receiving renal and lung transplantations (23–26). A recent study also showed CMV-induced clonal proliferation of γδ T-cells in patients after HSCT, demonstrating their importance in antiviral immunity (27). It is clear that this subset needs further investigation to elucidate its involvement in the context of our study and HSCT.

One month after infusion (3–5 weeks), there was a large heterogeneity in the frequency distribution between CD3+ and CD3- cells in peripheral blood between patients (*n* = 9, Figure 2A). In all analyzed patients except patient 7, the majority of CD3- cells consisted of NK-cells. In all analyzed patients except patient 10, the majority of CD3+ cells were conventional αβ T-cells. Parameters that determine this divergence remains to be determined. Despite not being significant, there seems to be a trend for a positive correlation between days after HSCT and cell infusion, and increasing amounts of αβ+ T-cells (Figure 2A). This is in agreement with the literature which favor an early reconstitution of the NK-cell compartment compared to conventional αβ T-cells (28, 29). All patients except patient 10, displayed a dominance of CD8+ T-cells which is also in line with previous literature regarding T-cell immune reconstitution after HSCT (30).

To conclude, this study has demonstrated the successful use of αβ T-cell depleted cell products as stem cell boosters in both pediatric (*n* = 1) and adult (*n* = 11) patients, with malignant (*n* = 8) and non-malignant (*n* = 4) underlying diseases suffering from primary graft failure and/or poor graft function with associated infections, poor immune reconstitution and/or mixed chimerism after their HSCT. These data exemplifies the future individualization of HSCT enabled by graft manipulation separation techniques. Larger studies are warranted to further assess the benefit of using αβ T-cell depleted cell products as stem cell boosters and study long-term outcome parameters.

## Author Contributions

MU, JM, MS, and ER designed the study. ER, MU, JT, and ST were responsible for performing the research. ER, JT, and ST were in charge of collecting the data. All authors (ER, MS, JT, ST, BÖ, PL, EW, JM, and MU) contributed to the analysis, interpretation, and writing of the manuscript.

### Conflict of Interest Statement

The authors declare that the research was conducted in the absence of any commercial or financial relationships that could be construed as a potential conflict of interest.
