# Supplementary material for: Individualization of Hematopoietic Stem Cell Transplantation Using Alpha/Beta T-Cell Depletion
Source: Front Immunol. 2019 Feb 11;10:189. doi: 10.3389/fimmu.2019.00189 (PMC6378311; doi:10.3389/fimmu.2019.00189)
Supplement: Supplementary file 1 [file Table_1.pdf]

Supplemental Table 1. Clinical definitions used in the study.

Category	Criteria/definition
**Infusion indications** (definitions of graft function according to stated EBMT criteria)	
Primary graft failure (GF)	ANC <0.5x10^9/L by day +28<br>Hemoglobine <80 g/L and platelets <20x10^9/L<br>RIC: Confirmation of donor cell origin is required<br>CBT: Up to day +42
Secondary graft failure	ANC <0.5x10^9/L after initial engraftment not related to relapse, infection or drug toxicity
Poor graft function (PGF)	Two or three cytopenias >2 weeks, after day +28 in the presence of donor chimerism >5%
Graft rejection (GR)	GF caused by immune rejection of donor cells mediated by host cells
**Transfusion dependency and G-CSF treatment** (decided by authors)	
Pre-infusion	Transfusions/doses given during the last month prior to αβ T-cell depleted infusion
Post-infusion	Transfusions/doses given day +30 to day +180 after αβ T-cell depleted infusion
**Overall clinical grading of severity of acute GVHD** (according to previous literature)	
Glucksberg, H., *et al*., Clinical manifestations of graft-versus-host disease in human recipients of marrow from HLA-matched sibling donors. Transplantation, 1974. 18(4): p. 295-304	

Abbreviations: ANC, absolute neutrophil count; RIC: reduced intense conditioning; CBT, cord blood transplantation.
