# Supplementary material for: Individualization of Hematopoietic Stem Cell Transplantation Using Alpha/Beta T-Cell Depletion
Source: Front Immunol. 2019 Feb 11;10:189. doi: 10.3389/fimmu.2019.00189 (PMC6378311; doi:10.3389/fimmu.2019.00189)
Supplement: Supplementary file 2 [file Table_2.pdf]

Supplemental Table 2. Major infectious problems among patients at the time of infusion of αβ T-cell depleted cell product and 3 months post infusion.

	Major infectious problems	
**Patient ID**	**At time of αβ T-cell depleted infusion**	**+ 3 months post-infusion**
**1**	Severe mucositis	None
**2**	Cerebral toxoplasmosis, CMV	Cerebral toxoplasmosis
**3**	Resistant HSV1-ulcer	Resistant HSV1-ulcer
**4**	CMV-enterocolitis, *Zygomycete**	CMV-enterocolitis, *Zygomycete**
**5**	None	None
**6**	CMV	None
**7**	CMV	None
**8**	*Aspergillus**	None
**9**	None	None
**10**	Recurrent *Clostridium difficile* (feces), HHV-6	Recurrent *Clostridium difficile* (feces), Influenza
**11**	Adenovirus (feces + blood), local *Staphylococcus aureus* (CVK)	None
**12**	CMV-colitis	CMV, *Pseudomonas* (septicemia x 2)

*lung lesions under treatment. *Abbreviations:* CMV, cytomegalovirus; HSV-1, herpes simplex virus 1; HHV-6, human herpesvirus 6; CVK, central venous catheter
