# Supplementary material for: Individualization of Hematopoietic Stem Cell Transplantation Using Alpha/Beta T-Cell Depletion
Source: Front Immunol. 2019 Feb 11;10:189. doi: 10.3389/fimmu.2019.00189 (PMC6378311; doi:10.3389/fimmu.2019.00189)
Supplement: Supplementary file 3 [file Table_3.pdf]

Supplemental Table 3. Normal reference values for platelets, granulocytes, lymphocytes and lymphocyte subsets in peripheral blood of healthy individuals.

**Age** (years)	**Platelets** (10^9/L)	**Granulocytes** (10^9/L)	**Lymphocytes** (10^9/L)	**CD3+** (cells/ µL)	**CD3+CD4+** (cells/ µL)	**CD3+CD8+** (cells/ µL)	**CD19+** (cells/ µL)	**CD16/56+** (cells/ µL)
1-2	150-400	1.5-8.8	3.0-11.2	N/A	N/A	N/A	N/A	N/A
3-4		2.0-9.6	2.1-9.6	N/A	N/A	N/A	N/A	N/A
≥18	F: 165-387<br>M: 145-348	1.6–5.9	1.1-3.5	780-2070	490-1340	190-800	90-400	70-420

Reference values according to Karolinska University Laboratory, Stockholm, Sweden.
